# Chlorhexidine Gel Use in the Oral District: A Systematic Review

**DOI:** 10.3390/gels5020031

**Published:** 2019-06-11

**Authors:** Luca Fiorillo

**Affiliations:** 1 Department of Biomedical and Dental Sciences and Morphological and Functional Imaging, Messina University, 98100 Messina, Italy; lfiorillo@unime.it or lucafiorillo@live.it; 2 Multidisciplinary Department of Medical-Surgical and Odontostomatological Specialties, University of Campania “Luigi Vanvitelli”, 80100 Naples, Italy

**Keywords:** chlorhexidine, oral surgery, mucosa healing, endodontics, prophylaxis, dentistry, chlorhexidine gel

## Abstract

Chlorhexidine compounds and their different formulations have been investigated several times, especially in the dentistry field. Chlorhexidine application for mouth rinsing immediately underwent oral contraindications, linked to the possibility of causing pigmentation to the teeth or relating to possible cytotoxic events after oral surgery. The positive effects, however, are considerable and its topical antiseptic action has been widely demonstrated by in vitro and clinical research. That’s the reason for its large application in different fields of dentistry. The aim of this study is to collect all the literature regarding the use of chlorhexidine gel in dentistry and all the numerous applications. The initial search on search engines obtained 232 results; then, following the application of the inclusion criteria there were 24 selected articles. The chlorhexidine gel appliance in the dental daily practice is direct to oral surgery, conservative endodontics, prevention and prophylaxis. The use of chlorhexidine has shown some positive effects, also in the case of systemic diseases prevention. Surely, this topical medicine used both professionally and prescribed for home use, can be considered a great help for the prevention of several oral pathologies with systemic implications too.

## 1. Introduction

### 1.1. Rationale

In recent years, the use of chlorhexidine has been increasingly common in different fields of medicine. Chlorhexidine is often present in different formulations such as mouthwashes, gels, galenic preparations, creams or dentifrices. The numerous compositions reflect the several applications and the different fields of use [1,2]. 

Chlorhexidine is a chemical synthesis disinfectant with a broad-spectrum antiseptic action, active against Gram-positive and Gram-negative bacteria and also against fungi [3,4,5,6]. It has a bactericidal action, drastically increasing the bacterial cell membrane permeability and altering the protein features; this causes the precipitation of macromolecules into the cytoplasm and subsequent cell death due to lysis of the bacterial cell or of the fungus. In the past, it was believed that it acted as a bacteriostatic, due to the inactivation of ATPases, preventing the replication of prokaryotic cells. Chlorhexidine, used in the form of a mouthwash, is often applied during dental visits in order to reduce oral bacterial flora. Furthermore, this can be used as prevention of and treatment for related plaque diseases, therefore some forms of gingivitis or periodontitis. By resolving these inflammations, it is also possible to limit gingival bleeding. The oral hygiene products made with chlorhexidine have bacteriostatic effects and are effective in reducing plaque, specifically they are able to limit the adhesion between the bacteria and the enamel, affecting the formation of the enamel film. The use of chlorhexidine gluconate has not been shown to reduce subgingival calculi [7] and in some studies it appears that they may even increase [7]. Chlorhexidine combined with xylitol induces a synergistic effect which increases its effectiveness. The role of chlorhexidine in preventing dental caries is controversial [8]. The data still disagree as regards the effect of chlorhexidine, and its uses in the form of mouthwash, on caries. The prevention of caries therefore does not seem to be related to the use of chlorhexidine. The same effects also concern chlorhexidine in gel and antiseptic solutions. Dental caries is a disease with a multifactorial etiology, it is currently appropriate to use fluoride applications, a correct diet and, above all, good oral hygiene practices as established. Furthermore, some recent research reports that chlorhexidine has an effect in reducing Streptococcus mutans, but in any case, the reduction of this bacterial strain or plaque seems to be unrelated to caries reduction. Use of chlorhexidine can cause stains on the teeth, particularly on the resin of the fillings [9,10]. Moreover, a sensation of alteration of taste (dysgeusia) may also occur; this last symptom can be avoided without prolonged use of chlorhexidine. Although there is not enough information in the literature to be able to state with certainty, some articles report that to maximize the effect of chlorhexidine it would be appropriate to wait at least 30 min for up to 2 h between brushing and using mouthwashes with this active ingredient. Chlorhexidine can also be used in the phases following oral surgery for the disinfection of surgical wounds as reported in the literature [11,12,13,14]. The history of the last twelve years (from the guidelines of the CDC 2002 onwards) is also the story of the progressive affirmation of 2% chlorhexidine in isopropyl alcohol (IPA) at 70% as a reference skin disinfectant for venous accesses. Both at the time of cutaneous antisepsis, due to the insertion of venous access and during dressing, 2% chlorhexidine in alcohol is the first choice for safe and efficient skin disinfection. It has long been known that the only antiseptics (= skin disinfectants) with real evidence of efficacy are 2% chlorhexidine, 70% alcohol and 10% iodopovidone. It has been known for some years that the combination of two of these antiseptics (i.e., 2% chlorhexidine in alcohol solution) is to be considered the best choice for skin disinfection before and after the placement of a venous access. New formulations have been developed subsequently [15].

### 1.2. Objectives

In the field of dentistry, an initial criticism has been created against this antiseptic because it could cause the formation of pigmentations on the enamel of the teeth, this is because chlorhexidine is able to attack the enamel film to remove it and thus makes the enamel susceptible to pigments in food or beverages. The purpose of this review is to evaluate in all the randomized studies on the advantages or disadvantages of the use of chlorhexidine and above all, which and how many fields it is used in [16]. The function of the article wants to be more than that of clarifying all the advantages and defects of the topical oral cavity antiseptic but also of knowing the multiple uses.

## 2. Results and Discussion

### 2.1. Synthesis of Results

**Figure 1:**
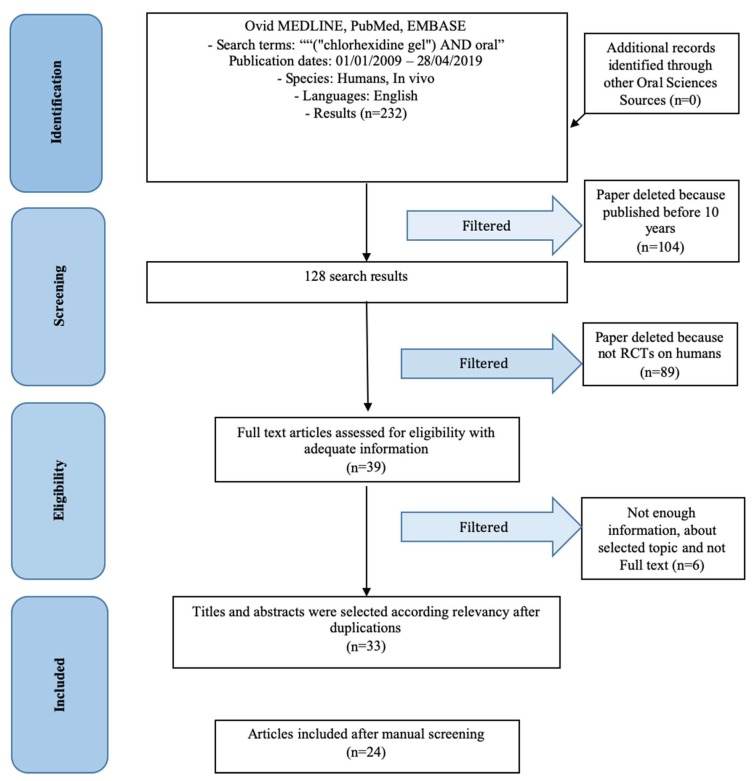
PRISMA Flow chart.

Using the search parameters expressed in the materials and methods section, it was possible to filter the results until 24 jobs was obtained (Figure 1). These works have been screened by the author and subsequently analyzed individually in order to bring to light their salient features. The articles have different characteristics between them and not all of them relate the use of chlorhexidine with other topical antiseptics or in the same areas of use, so it is not possible to make a univocal statistic. In Table 1 it is possible to closely analyze some results. The search terms were used in the scientific databases on 01 May 2019. 

It is necessary to report that some articles, despite dealing with the topic of the study, do not fit our topic precisely. Some of the resulting articles deal with modified chlorhexidine-based gels or with samples that have pathologies or with experimental treatments [17,18,19].

### 2.2. Summary of Evidence

As can be seen from the results, the uses of chlorhexidine are multiple, often this is used following oral surgery or for the treatment of periodontal or peri-implant lesions [47]. Certainly, it is also necessary to mention the reasons for prevention and prophylaxis. This is to be considered useful above all in patients suffering from systemic diseases that can hardly face dental treatments. Prevention and therefore the reduction of treatments [48,49,50], especially the complex ones, would be an advantage for these patients, such as the syndromic ones [38,39,51,52,53]. From the chemical point of view, chlorhexidine is a cationic biguanide that is poorly soluble in water. To make it water-soluble, the substance has to be combined (or rather, salified) with gluconic acid: not by chance, in the preparation of the mouthwash, the compound is in the form of chlorhexidine digluconate. Chlorhexidine acts in two ways:

- Powerful bactericide: Chlorhexidine alters the protein structure of the bacterial cell membrane: by exaggeratingly increasing its permeability, the substance promotes the precipitation of cytoplasmic proteins and the consequent cell death by bacterial lysis.
- Bacteriostatic: Once it was believed that chlorhexidine could block the replication of bacteria; today it is well known that it is also able to kill them.

According to Coello-Gomez et al. [20] there are no significant differences between the use of chlorhexidine gel or SOS in pain, swelling or Visual Analogue Scale (VAS) scale after a third molar surgery. According to Sinjari et al. [21] the use of chlorhexidine gel inside the fixture/abutment of a dental implant connection can reduce the peri-implant marginal bone loss. This is an important result; in fact it makes us aware of the fact that the loss of marginal peri-implant bone is due to the presence of bacteria in the implant connection [47,54,55]. Despite the bioengineering efforts, the connection always presents a gap. The fusion of titanium or the switching platform was not enough to reduce this slow and constant phenomenon. Improving this interface could eliminate the problem of marginal bone loss, chlorhexidine in gel is an excellent technique [21]. According to Rusu et al. [22], both soluble chlorhexidine gel and gingiva-adhering chlorhexidine gel present an improvement of clinical parameters after scaling and root planning compared to no topical therapy. Rubio-Palau et al. [23] evaluated a reduction of 22% of alveolar infection after dental extraction, with 0.2% chlorhexidine application compared to placebo, when chlorhexidine gel was used. This could be a future protocol perspective for post-extractive alveolus [50]. Levin et al. [24] suppose that the use of a water jet mixed with chlorhexidine can be useful to prevent, and as therapy for, periimplantitis lesions. According to Jesudasan et al. [25], alveolar osteitis with pain or inflammation can be reduced following a 0.2% chlorhexidine gel protocol or a eugenol based paste protocol after third molar surgery. Haraji et al. [26] in a 2015 Randomized Controlled Trial (RCT), found that the use of chlorhexidine gel after surgery, third molar surgery, could reduce post-operative pain. This was a surprising result, because a drug like chlorhexidine, which has no systemic contraindications and does not present a heavy absorption and metabolism for the patient, can replace a systemic pain-relieving or anti-inflammatory therapy which is often rather burdensome to the systemic load, such as the liver or the kidneys [56]. According to the evaluated literature, chlorhexidine gel did not show side effects. Freudenthal et al. [27] concluded that there are statistically important differences in alveolar osteitis between chlorhexidine and non-use after a third molar surgery. According to Diaz-Sanchez [28], chlorhexidine gel 0.2% did not contribute clinical improvement in patients undergoing radiation therapy and chemotherapy [52]. In the second evaluated study by Haraji et al. [29], an intra-alveolar application of chlorhexidine gel could contribute to a lower risk of dry socket. Singh et al. [30] show the endodontical properties of chlorhexidine. Dressed canal with chlorhexidine and chlorhexidine + calcium hydroxide compared to no dressing or just calcium hydroxide gave rise to less pain. According to Pukallus et al. [31] using 0.12% chlorhexidine gel and 304% fluoride toothpaste can prevent early childhood caries. Lima et al. [32] 1% chlorhexidine gel compared to calcium hydroxide/camphorated paramonochlorophenol presented lower success rate on intracanal treatment against anaerobic bacteria and S. mutans. According to De Siena et al. [33], chlorhexidine treatments (0.2% mouthwash or 1% gel) is beneficial to the treatment of peri-implant mucositis. De Lucena et al. [34] show how both chlorhexidine gel and octenidine gel are useful intracanal medicaments. Almeida et al. [35] evaluated the differences between chlorhexidine gel and NaOCl, used like a canalar irrigant, and found no differences. According to Heitz-Mayfield et al. [36] chlorhexidine gel can be useful against mucositis after non-surgical debridement. Torres-Lagares et al. [37] showed that an intra-alveolar application of bioadhesive 0.2% chlorhexidine gel seems to reduce the incidence of alveolar osteitis. Slot et al. [40] concluded that the use of chlorhexidine 1% gel gives better results than 0.12% toothpaste or compared to normal dentifrices in inhibiting plaque accumulation. Lopez-Jornet et al. [41] concluded that the application of polyvinylpyrrolidone sodium hyaluronate and chlorhexidine digluconate decreases the symptoms after oral surgery like an oral mucosa biopsy. Cabov et al. [42] evaluated patients in an intensive-care unit. Among surgical intensive care unit patients, oral decontamination with chlorhexidine significantly decreased colonization in other districts. According to Paolantonio et al. [43], xanthan-based chlorhexidine gel can be used during scaling and root planning with better outcomes. Malkhassian et al. [44] said that canal disinfection with chlorhexidine is comparable to NaOCl. According to Hauser-Gerspach et al. [45] the application of ozone therapy or in any case of chlorhexidine-based gel in the case of deep caries did not show an immediate antimicrobial effect, if the infected dentin was not removed. Gomes et al. [46] show how both NaOCl and Chlorhexidine gel 2% were not effective in eliminating endotoxins from a root canal. The different fields of use of chlorhexidine and those which are the results of the last 10 years present in the literature have been highlighted.

### 2.3. Additional Analyses

The molecule, poorly soluble in an aqueous solution, has a double charge as a salt and behaves like a positive cation, generally associated with a pair of water-soluble negative anions such as chloride, acetate or gluconate (anion of gluconic acid); moreover, it is a symmetric molecule that contains two strongly apolar benzene groups. It is commercially available for various applications, such as chloride or acetate, for medical applications digluconate is usually used (gluconic acid is naturally present in fruit, honey and wine, it is added as an additive as an acidity regulator, it is also used in cleaning products). Chlorhexidine as such is a practically apolar organic compound and therefore poorly soluble in water but also in poorly polar organic solvents such as dichloromethane (CH_2_Cl_2_). On the contrary, the gluconate anion is soluble in water and is therefore an excellent counter-ion for the counter-chlorhexidine, making the compound moderately water-soluble (max 0.5–2.0% at a pH close to neutrality). It is inactivated by sodium lauryl sulphate (SLS, NaLS, LSS) and by triclosan, both possible components of toothpaste, which is why chlorhexidine treatment should be performed away from tooth cleaning with toothpaste. Chemically chlorhexidine is a bis-biguanide and is available as a counter-cation generally associated with three counter-anionic species such as: acetate, chloride and gluconate; the latter is the most used form in dentistry. Globally, the organic chlorhexidine molecule is therefore neutral, apolar and of medium-large dimensions (due to the strongly apolar benzene groups and arranged at the extremes of the molecule which occurs mainly in linear or unfolded form) and this explains its poor direct solubility in water. Chlorhexidine is deactivated by all ionic compounds, such as some anionic compounds present in dentifrices. For this reason, it can be seen from the literature that low chlorhexidine products should be used at least 30 minutes after normal oral/dental hygiene products. Its effectiveness can also be influenced by nutrition, so it is good to avoid civi or drinks for at least an hour. Furthermore, the function of chlorhexidine is influenced by the presence of blood or inflammatory fluid or soaps. Studies show that only 30% of the active ingredient of chlorhexidine-based products remains present in the oral cavity [57,58,59,60,61].

Chlorhexidine gluconate is poorly absorbed by organisms. According to the literature, chlorhexidine is slightly absorbed from the gastrointestinal tract in human and animal subjects. The average plasma level of chlorhexidine gluconate reaches a blood peak of 0.206 mg/g in humans 30 min after ingesting a 300 mg dose of the drug. Detectable levels of chlorhexidine gluconate, 12 h after its administration, were no longer present in the plasma of the subjects to whom it was administered. It does not undergo any metabolism and the excretion of chlorhexidine gluconate occurs mainly with feces (~90%). In the urine, less than 1% of chlorhexidine gluconate ingested was excreted. The remarkable antibacterial properties of chlorhexidine are due to the ability to alter the structure of the cell membrane with consequent precipitation of cytoplasmic proteins. It has a greater antibacterial effect on Gram-positive cocci and lesser on Gram-negatives [62]. It also has moderate activity against pericapside viruses, non-enveloped bacteria, while viruses and spores are resistant [63]. Clinically it has the advantage of a long duration of action on the teeth and on the oral mucosa without being absorbed by the same mucosa. Furthermore, chlorhexidine is 100% excreted not metabolized. It acts only and selectively on the cell membrane of prokaryotes. Chlorhexidine has an important property: substantivity; this property ensures that this antiseptic remains bound in soft and hard tissues for 8–12 h, allowing a useful pharmacological action in the oral cavity environment. It is used as an active ingredient in mouthwashes to prevent the formation of dental plaque and to reduce its pathogenicity; it also promotes inhibition and delays the eventual development of gingivitis and effectively fights halitosis. The greater anti-plaque action compared to other antiseptics, the broad-spectrum bactericidal action, the substantivity and non-induction in the oral cavity of bacterial resistance, makes it prefer to other active principles, avoiding among other things the development of resistant bacteria and difficult to treat with periodontal pharmacotherapy.

Chlorhexidine gluconate, in oral rinsing, acts as an antimicrobial throughout the duration of the rinse; the clinical significance of this antimicrobial activity in oral rinsing is not entirely clear. Some microbiological tests have shown a general reduction of the bacterial load with the use of chlorhexidine. Tested, aerobic and anaerobic bacteria were reduced from 54 to 97% after a use of 6 chlorhexidine drugs. No bacterial resistance has been shown following the use of chlorhexidine, nor for the realization of opportunistic infections or negative characteristics for the oral microbiota. [64,65]. After three months of use of chlorhexidine gluconate in oral rinsing and then his stop, the number of plaque bacteria returned to basal levels.

**Figure 2:**
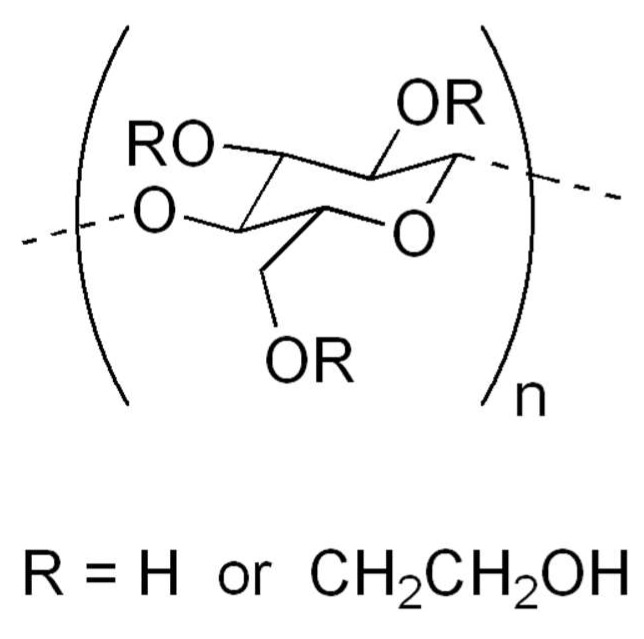
Hydroxyethylcellulose gelificant.

**Figure 3:**
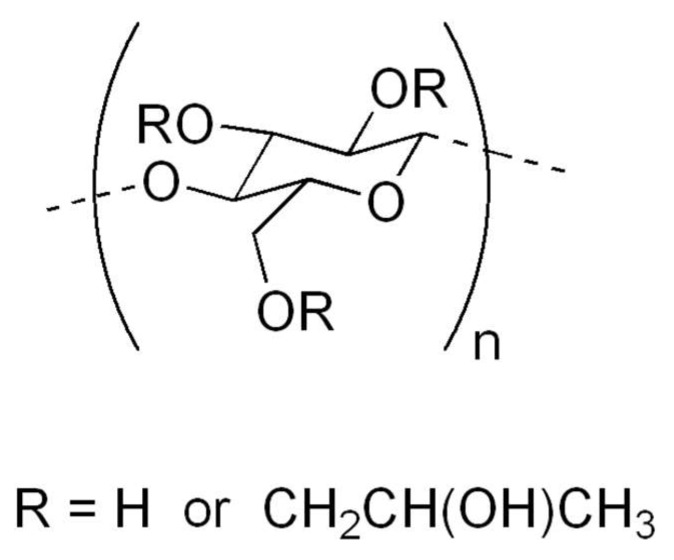
Hydroxypropyl cellulose gelificant.

Chlorhexidine for this reason is used in different fields of medicine, such as dermatology, dentistry, gynecology, urology [59,63,66,67,68,69,70,71,72]. It is also used in veterinary medicine or in hygiene, for the disinfection of environments, materials and of operators [73]. Chlorhexidine-based gels contain, so that they can maintain the gel form of gelling substances. These substances, in addition to keeping the gel in place, prevent it from being swallowed; moreover, they are often associated with other compounds with mucoadhesive characteristics. Chlorhexidine-based gels can often contain the following inactive compounds: methylparaben, propylparaben, Hydroxypropylcellulose, hydroxyethylcellulose, sodium hydroxide, propylene glycol, Macrogolglycerol hydroxystearate, Sodium acetate, different essential oils, Isopropyl alcohol and certainly purified water. Among these, those with gelling or thickening properties are different. Hydroxyethylcellulose (Figure 2) is a derivative of cellulose which differs from the latter in that the hydroxyl groups of the polymer have been replaced by hydroxyethyl groups, which prevent it from becoming crystallized. It is highly hydrophilic and has thickening and gelling properties. It can be more or less soluble in water depending on the length of the polymer chains of which it is made. Polar liquids such as water and hydroxyethylcellulose form a gel which is the basis for the preparation of creams and gels for cosmetic or external use. It is a component of the solutions used to humidify contact lenses. It is used in the emulsion polymerization process as an emulsifier or stabilizer. Or they may contain hydroxypropyl cellulose, which is a derivative of cellulose, soluble in cold water and in various organic solvents, obtained by treatment with propylene oxide. The polymer has the formation of ether bonds, with the introduction of hydroxypropyl groups which are generally present in a variable ratio from 0.02 to 0.3 moles per monomer unit. It should be noted that the hydroxypropyl groups add a further –OH which is also capable of reacting, therefore, theoretically, the substitutable hydroxyls per unit of monomer are greater than 3. The addition of ether bonds in the cellulose molecule increases their solubility in water, while introducing groups of lesser polarity. Hydroxypropyl cellulose (Figure 3) produces gums that possess a certain surface activity, making it useful for stabilizing emulsions and foams. Hydroxypropyl cellulose is used in the pharmaceutical industry as a binder in the formulation of tablets and in ophthalmological preparations for dry eyes. It is also a food additive known as E463. Rheology is the science that studies the deformation behavior and the flow of matter when subjected to stress in specific thermodynamic conditions for a period of time. Based on the behavior of each fluid, it can be classified into two large groups: Newtonian fluid, when the viscosity of the material is equal, regardless of the shear stress (force applied in a given area of the fluid) applied to a given fluid temperature and fluids non-Newtonian, which are divided into time dependent, time-independent and viscoelastic. In order to produce the chlorhexidine-based gel used in the pharmaceutical field, the gelling agent (a cellulose derivative) is dissolved in hot water under agitation. Once the gel is formed, the 1% solution of chlorhexidine digluconate is added and the formulation is left under stirring for 24 h. To obtain a good mucoadhesiveness, it is necessary to optimize the polarity of the polymeric surface and the mobility of the chains based on the glass transition temperature and the contact angle of the water polymer. The use of mucoadhesive polymers allows the release of oral active ingredients to be controlled. In any case, these polymers should be flexible and small in size to follow the movements of the cheeks and not cause irritation. Hydrogen ions are suitable for creating mucoadhesive systems thanks to their flexibility and biocompatibility. The force that determines adhesion is promoted by the degree of similarity between the surface polarity of hydrogen and that of the substrate, in fact, it is the surface polarity that determines the inter-polymer-mucosa interfacial bonds. The mobility of the polymer chains allows the interpenetration of the polymer itself in the mucous layer. The glass transition temperature can be used to measure the mobility of the chains. The percentages of gelling agent may be different depending on the gel considered. The different formulations of chlorhexidine-based gel have a good solubilizing efficacy against the active ingredient. For example, gels containing 3% carboxymethylcellulose (CMC) and 3% hydroxypropyl methylcellulose (HPMC) have a lower solubility of chlorhexidine than those composed of only 3% hydroxypropyl cellulose (HPC). The chlorhexidine with different chain fatty acids gels have also good solubility values of chlorhexidine. In this case, it is possible to observe that the two polymers containing the lauric acid as a hydrophobic substituent, have the two highest solubility values. Observing the gels obtained from the combinations of two different bioadhesive polymers used in the mixture, it is observed that all the combinations of the polymers cause an increase in the solubility of the active principle in the developed formulation. This result can be explained in relation to the capacity possessed by the carboxymethylcellulose to bind the drug in a stable manner, through electrostatic interactions and thus to effectively control the release. Carboxymethylcellulose is in fact present in all three gels that have the slowest dissolution rate, even if in different percentages. Instead, from the dissolution profiles of chlorhexidine from the developed mucoadhesive tablets it emerges that these are formulations able to effectively control the dissolution and consequently the release of the drug in the oral cavity. It is known that both Carbopol 974P and HPMC have good mucoadhesive properties and furthermore their polymer swelling mechanism, following contact with the aqueous environment, makes them able to carry out an equally effective release control. of the active ingredient. The best bioadhesivity is possessed by gels made up of 3% of carboxy methylcellulose and 2% of hydroxypropyl cellulose. This result is the one that has the best bio-adhesion characteristics, two of the known mucoadhesive properties of the carboxymethyl cellulose. Although the hydroxypropyl methylcellulose is used in combination with 2% of carboxy methylcellulose, it is able to confer good mucoadhesive capacity to the formulation. It emerges that the carboxy methylcellulose combined with the other cellular derivatives results in the bioadhesive properties of the formulations developed. The release profiles of the 3% CMC are those that guarantee the highest release of active ingredients in the oral cavity, associated with a controlled mucosal permeation. This result is also based on the presence of a single polymer as a mucoadhesive excipient, which has a positive effect on the development of the polymeric matrix as a result of swelling. The others, with two different bioadhesive polymers, combined with each other in different percentages, show released profiles for each other and associated with controlled mucosal permeations. 

### 2.4. Limitations

The study takes into consideration a considerable number of jobs and many of these have a low risk of bias. Unfortunately, the works taken into consideration, however, cannot be related to each other and often chlorhexidine is compared to other materials and used in different formulations and dosages.

## 3. Conclusions

From what can be seen from the results, chlorhexidine is used in different areas of dentistry. It is used for the treatment of mucositis, peri-implantitis, for the prevention of alveolitis or for conservative or endodontic treatment. The results are clearly in favor of the use of chlorhexidine rather than nothing, despite this there are also other oral cavity topical antiseptics that have similar effects to chlorhexidine. A unique fact cannot yet emerge from this study but the positive effects of chlorhexidine are well demonstrated.

## 4. Materials and Methods 

### 4.1. Protocol and Registration 

This review is in PROSPERO database with ID number 233453. It is an international database of prospectively registered systematic reviews. 

### 4.2. Focus Question

The following focus question was developed according to the population, intervention, comparison and outcome (PICO) study design.

For dental patients, does the use of chlorhexidine gel reduce the future risk of complications compared with other topical oral antiseptics?

And as alternative question:

Do dental patients, who use chlorhexidine gel, have a decreased risk of oral and post-surgical complications?

### 4.3. Information Sources

The search strategy incorporated examinations of electronic databases, supplemented by hand searches. A search of PubMed and Dentistry and Oral Sciences Source [74,75], for relevant studies published in the English language.

### 4.4. Search

The keywords used in the search of the selected electronic databases included the following: 

“(“chlorhexidine gel”) AND “oral”

Keywords was selected by author with the aim to collect all articles about this topic.

### 4.5. Selection of Studies

All results, inclusion and exclusion criteria have been verified by the author. For the stage of reviewing of full-text articles, a revision was performed.

### 4.6. Types of Selected Manuscripts

The review included studies on humans published in the English language. Letters, editorials and PhD theses were excluded. 

### 4.7. Types of Studies

The review included all human RCTs about use of chlorhexidine gels in different formulations.

### 4.8. Inclusion and Exclusion Criteria

The full text of all studies of possible relevance was obtained for assessment against the following inclusion criteria:

- Chlorhexidine gel use in randomized clinical trials (RCTs)
- Roles of chlorhexidine gel
- Human RCTs

The applied exclusion criteria for studies were as follows:

- Studies involving patients with other specific diseases, immunologic disorders or other oral risk related systemic conditions
- Not enough information regarding the selected topic
- No access to the title and abstract in English language
- Not older than 10 years RCTs

### 4.9. Sequential Search Strategy

After the search and the exclusion of all the non-relevant articles thanks to the search filters, a manual review of the resulting articles was conducted. Articles that did not contain sufficient information were removed and the full text was read to highlight whether or not there was sufficient information to support the review. Articles more than 10 years old have been excluded.

### 4.10. Data Extraction

The data were independently extracted from studies in the form of variables, according to the aims and themes of the present review, as listed onwards.

### 4.11. Data Collections

Data were collected from the included articles and arranged in the following fields (Table 1):

- “Author (Year)”—Revealed the author and year of publication
- “Features”—Results and features evaluated about chlorhexidine gel
- “Field”—Field of use of chlorhexidine gel
- “Statistics”—Significative or not results
- “Type”—Type of article

### 4.12. Risk of Bias Assessment

Assessment of risk of bias was undertaken during data extraction process. The Cochrane tools were used to carry out the bias risk assessment of the items considered. The level of bias risk, in accordance with the above, was defined as low, moderate, high or unclear [76]. Bias is assessed as a judgment (high, low or unclear) for individual elements from five domains (selection, performance, attrition, reporting and other). Data are shown in Table 2.

## Figures and Tables

**Table 1:** Results of the review.

Author (Year)	Features	Field	Statistics	Type
Coello-Gomez et al. (2018) [20]	Chlorhexidine gel vs. mouthwash with super-oxidized solution (SOS)	Oral surgery	Not significative	RCT double blinded
Sinjari et al. (2018) [21]	Chlorhexidine gel vs. placebo gel for periimplantitis prevention	Implantology; Periodontology	Significative	RCT blinded
Rusu et al (2017) [22]	Chlorhexidine-based gingiva-adhering gel containing herbal ingredients vs. 1% chlorhexidine water-soluble gel for periodontitis prevention after scaling	Periodontology	Not significative	RCT blinded
Rubio-Palau et al. (2015) [23]	0.2% Chlorhexidine gel vs. placebo for alveolar osteitis prevention after third molar surgery	Oral surgery	Not Significative	RCT double blind
Levin et al. (2015) [24]	Chlorhexidine gel adjunct water jet for periimplantitis prevention	Implantology; periodontology	Not Significative	RCT
Jesudasan et al. (2015) [25]	0.2% Chlorhexidine gel vs. eugenol based paste vs. control for alveolar osteitis prevention after third molar surgery	Oral surgery	*P* = 0.002 Eugenol paste has better results	RCT
Haraji et al. (2015) [26]	0.2% Chlorhexidine after third molar extraction for alveolitis prevention	Oral surgery	Significative, chlorhexidine can reduce pain	RCT split mouth
Freudhental et al. (2015) [27]	0.2% Chlorhexidine vs. placebo for alveolar osteitis prevention	Oral surgery	Not significative	RCT double blinded
Diaz-Sanchez et al. [28]	Bioadhesive 0.2% chlorhexidine gel vs. placebo for mucositis radio and chemotherapy induced prevention	Oral surgery; periodontology	Not significative	RCT double blinded
Haraji et al. (2014) [29]	0.2% Chlorhexidine gel vs. control for dry socket (DS) prevention after third molar surgery	Oral surgery	*P* = 0.004 Use of chlorhexidine lowered DS	RCT split mouth
Singh et al. (2013) [30]	Calcium hydroxide paste mixed with 2% chlorhexidine gel vs. 2% chlorhexidine gel, vs. calcium hydroxide paste vs. control (no dressing) for intracanal medications	Endodontics	Group I and II *P* < 0.05	RCT double blinded
Pukallus et al. (2013) [31]	0.12% Chlorhexidine (CHX) gel vs. 304% fluoride toothpaste to prevent early childhood caries	Prophylaxis	Not significative	RCT
Lima et al. (2013) [32]	1% Chlorhexidine gel vs. calcium hydroxide/camphorated paramonochlorophenol (Callen PMCC) vs. a one-visit endodontic treatment to bacterial proliferation	Endodontics	Chlorhexidine vs. mutans streptococci *P* = 0.10; PMCC vs. anaerobic bacteria *P* = 0.002	RCT split mouth
De Siena et al. (2013) [33]	1% Chlorhexidine gel vs. 0.2% chlorhexidine for peri-implant mucositis treatment	Implantology; Periodontology	Not significative	Observational study
De Lucena et al. (2013) [34]	Calcium hydroxide paste (CH) vs. chlorhexidine gel (CHX-gel) (5.0%) vs. chlorhexidine/gutta-percha points (CHX-GP) vs. octenidine gel (OCT-gel) (5.0%) for dentin E. faecalis contamination preventing	Endodontics; Restorative dentistry	CHX-gel and OCT-gel significative	RCT
Almeida et al. (2012) [35]	5.25% Sodium hypochlorite (NaOCl) or 2% chlorhexidine gel (CHX) for apical periodontitis preventing	Endodontics; Periodontology	Not significative	RCT
Heitz-Mayfield et al. (2011) [36]	0.5% Chlorhexidine gel vs. placebo gel for peri-implant mucositis managing	Implantology; Periodontology	Significative	RCT double blinded
Torres-Lagares et al. (2010) [37]	0.2% Chlorhexidine gel vs. placebo for postextractive alveolitis prevention after third molar extraction on bleeding disorders patients [38,39]	Oral surgery	Significative	RCT double blinded
Slot et al. (2010) [40]	1% Chlorhexidine gel vs. 0.12% chlorhexidine dentifrice-gel vs. control dentifrice vs. 0.2% chlorhexidine mouthwash for plaque formation prevention	Prophylaxis	1% Chlorhexidine gel and 0.2% chlorhexidine mouthwash were significative	RCT
Lopez-Jornet et al. (2010) [41]	Polyvinylpyrrolidone-sodium hyaluronate (Aloclair) gel vs. 0.2% chlorhexidine digluconate gel vs. control for symptom prevention after mucosa biopsy	Oral surgery	Significative	RCT
Cabov et al. (2010) [42]	Chlorhexidine gel vs. control to prevent oral mucosa contamination	Oral surgery; Prophylaxis	Significative	RCT double blinded
Paolantonio et al. (2009) [43]	Chlorhexidine gel vs. Xantan base chlorhexidine	Oral surgery; prophylaxis	Significative	RCT
Malkhassian et al. (2009) [44]	BioPure MTAD vs. 2% Chlorhexidine gel for root canal treatment	Endodontics	Not significative	RCT double blinded
Hauser-Gerspach et al. (2009) [45]	Gaseous ozone and chlorhexidine gel for cavities prevention	Prophylaxis	Not significative	RCT
Gomes et al. (2009) [46]	2.5% Sodium hypochlorite (NaOCl) vs. 2% chlorhexidine (CHX) gel on eliminating oral bacteria	Endodontics; Prophylaxis	Not significative	RCT

**Table 2:** Risk of bias table (High +, Low −, Unclear ?).

Author (Year)	Random Sequence Generation	Allocation Concealment	Selective Reporting	Other Sources of Bias	Blinding	Incomplete Outcome Data
Coello-Gomez et al. (2018) [20]	-	-	-	?	-	-
Sinjari et al. (2018) [21]	?	?	-	?	+	-
Rusu et al (2017) [22]	-	-	-	-	-	-
Rubio-Palau et al. (2015) [23]	-	-	-	?	-	-
Levin et al. (2015) [24]	-	-	-	-	+	-
Jesudasan et al. (2015) [25]	+	-	-	-	+	-
Haraji et al. (2015) [26]	-	-	-	?	+	-
Freudhental et al. (2015) [27]	-	-	-	?	-	-
Diaz-Sanchez et al. [28]	-	-	-	-	-	-
Haraji et al. (2014) [29]	-	+	-	-	+	-
Singh et al. (2013) [30]	-	-	-	?	-	-
Pukallus et al. (2013) [31]	-	-	-	-	+	-
Lima et al. (2013) [32]	-	-	-	?	+	-
De Siena et al. (2013) [33]	-	-	-	-	+	-
De Lucena et al. (2013) [34]	-	-	-	-	+	-
Almeida et al. (2012) [35]	-	-	-	-	+	-
Heitz-Mayfield et al. (2011) [36]	-	-	-	?	-	-
Torres-Lagares et al. (2010) [37]	-	-	-	-	-	-
Slot et al. (2010) [40]	-	-	-	?	-	-
Lopez-Jornet et al. (2010) [41]	+	-	-	-	+	-
Cabov et al. (2010) [42]	-	-	-	?	-	-
Paolantonio et al. (2009) [43]	-	-	-	?	+	-
Malkhassian et al. (2009) [44]	-	-	-	?	-	-
Hauser-Gerspach et al. (2009) [45]	-	-	-	?	+	-
Gomes et al. (2009) [46]	-	-	-	?	+	-

## Abbreviations

NaOCL	sodium hypochlorite
CHX	chlorhexidine
DS	Dry socket
